# Preterm Parturient with Polyhydramnios and Pancreatitis: Primary Presentation of Hyperparathyroidism

**DOI:** 10.1155/2018/2091082

**Published:** 2018-01-21

**Authors:** Esther S. Han, Katherine Fritton, Phoebe Bacon, Martin K. Slodzinski, Cynthia Argani

**Affiliations:** ^1^Department of Gynecology and Obstetrics, Johns Hopkins University School of Medicine, Baltimore, MD, USA; ^2^Division of Obstetric, Gynecologic and Fetal Anesthesiology, Johns Hopkins University School of Medicine, Baltimore, MD, USA

## Abstract

**Objective:**

To report a case of severe hypercalcemia secondary to primary hyperparathyroidism in a late-preterm pregnant patient and review medical and surgical treatments as well as obstetric and neonatal outcomes.

**Background:**

Diagnosis of parathyroid disease during pregnancy can be difficult due to nonspecific presentation. Management decisions are complex and require multidisciplinary collaboration.

**Case:**

A 29-year-old G2P1001 woman at 35 weeks and 3 days' gestation presented with preterm contractions, polyhydramnios, pancreatitis, and severe hypercalcemia. Work-up revealed primary hyperparathyroidism with multiple thyroid nodules. Patient history, presentation, and biopsy were suspicious for parathyroid carcinoma. Despite severe hypercalcemia, both patient and fetus remained stable and medical management was pursued in an attempt to optimize mother and fetus prior to delivery. Due to recalcitrant hypercalcemia, surgical resection was ultimately required. She was subsequently delivered in the setting of preterm labor. Final pathology revealed parathyroid adenoma with atypia and occult papillary thyroid carcinoma.

**Conclusion:**

Symptoms of hypercalcemia can mimic those of a normal third trimester pregnancy and can have serious maternal and fetal effects if left untreated. A coordinated, multidisciplinary approach to these patients is necessary.

## 1. Introduction

Hypercalcemia is a rare but dangerous condition in pregnancy with significant maternal and fetal implications. Most cases of hypercalcemia reported in the literature are due to primary hyperparathyroidism; however, diagnosis can be difficult as symptoms associated with hypercalcemia are familiar in pregnancy, including constipation, nausea, vomiting, epigastric pain, fatigue, polyuria, and vague musculoskeletal discomforts of the back, hips, and joints [1]. One case series identified five cases of maternal hypercalcemia diagnosed with otherwise unexplained fetal polyhydramnios [2]. Most cases do not cause hypercalcemic crisis [1].

Management of maternal disease and delivery planning must take into account the implications for both mother and fetus. In cases of severe hypercalcemia in the third trimester, the initial reaction may be to deliver the fetus; however, disruption of transplacental calcium shunting can have significant consequences. Delivery may lead to a precipitous increase in maternal serum calcium levels, as calcium that was previously shunted to the fetus now remains in maternal circulation [1, 3] Additionally, in the absence of maternal calcium, the neonate may experience a significant hypocalcemic crisis as neonatal PTH levels are suppressed and the previously maternally supplied calcium is abruptly removed [3–5]. Even in the case of severe hypercalcemia, the acute maternal presentation, concern for malignancy, fetal clinical status, maternal medical and surgical history, and response to medical management should all be taken into careful consideration when planning antepartum and intrapartum management. Medical optimization and timing of surgical intervention as well as fetal monitoring and postoperative care require interdisciplinary collaboration.

## 2. Case Presentation

The patient is a 29-year-old G2P1001 Hispanic woman with uncertain last menstrual period and no previous prenatal care who presented to Labor and Delivery for evaluation of preterm labor. She complained of nausea, vomiting, and acid reflux, which she had experienced throughout this pregnancy. Obstetric history included a prior full term Cesarean delivery without complications. Medical history revealed a history of kidney stones and pancreatitis. Surgical history was remarkable for cholecystectomy, appendectomy, and ureteral stone removal. All previous medical and surgical care took place in Honduras and no records were available.

Ultrasound established an estimated gestational age of 34 weeks with bilateral echogenic fetal kidneys. Amniotic fluid was normal with an amniotic fluid index (AFI) of 15.4. She was found to have contractions and her cervical exam changed from closed to 1 cm dilated. Given concern for preterm delivery, she was given a dose of betamethasone in accordance with recently published Antenatal Late Preterm Steroids trial demonstrating benefit between 34 and 36 weeks and 6 days' gestation [6]. Her contractions subsequently decreased and a repeat cervical exam was unchanged. She was discharged home with instructions to return in 24 hours for her second dose of betamethasone, but she did not return the following day.

Seven days later, the patient returned to Labor and Delivery with contractions and diffuse abdominal pain. She reported vague and worsening discomfort across the entirety of her abdomen. She also complained of nausea, vomiting, and worsening acid reflux. She denied sharp or radiating epigastric pain, flank pain, or dysuria but noted polyuria, fatigue, and weakness. Her vital signs were within normal limits. Tocodynamometry demonstrated uterine irritability and contractions; however, her cervical exam remained unchanged from her evaluation one week before. Fetal heart tracing had moderate variability without acceleration. Ultrasound showed new polyhydramnios with an AFI of 41.3 with a biophysical profile of 8/10.

Despite thorough and repeat history and physical exams, no focal signs or symptoms concerning for a specific etiology of her discomfort were elucidated. Her diffuse discomfort was attributed to significant uterine distension from polyhydramnios and GERD.

However, labs demonstrated severe hypercalcemia, pancreatitis, and acute kidney injury: a corrected serum calcium of 16.9 mg/dL (ionized calcium 2.05), hyponatremia, hypochloremia, creatinine 1.4 mg/dL, BUN 41 mg/dL, amylase 334 U/L, lipase > 3000 U/L, WBC 15.8 K/mm^3^, and Hgb 9.7 g/dL. AST and ALT were within normal limits. An EKG was performed that showed ST elevations consistent with hypercalcemia. Troponins were negative. Focused physical exam was remarkable for a palpable thyroid gland (bilateral lobes) without rubor, calor, dolor, or bruits. Neurologic exam showed no focal deficits and full strength in bilateral upper and lower extremities. The patient remained alert and oriented × 3. Additional labs were drawn and were significant for low TSH (0.02 ng/dL), elevated free T4 (1.9 ng/dL), and significantly elevated PTH (937 pg/mL).

Thyroid ultrasound showed bilateral subcentimeter nodules and a 2.7 cm hypoechoic noncalcified solid lobulated nodule in the right thyroid lobe. A smaller, 0.7 cm nodule was also seen in the lower pole of the left thyroid lobe. A 0.6 cm predominantly cystic nodule was seen in the junction of the isthmus and right thyroid lobe. Abdominal ultrasound showed no dilatation of the intrahepatic biliary tree with a normal common bile duct. Florid bilateral nephrocalcinosis was present, consistent with longstanding hypercalcemia. Biliary etiology of her pancreatitis was deemed unlikely.

Fetal status remained reassuring with continuous monitoring. Aggressive IV fluid hydration was initiated with immediate improvement in pancreatitis but with minimal improvement in calcium levels. Given the patient's young age, severe hypercalcemia, very high PTH levels, and neck mass, there was concern for parathyroid cancer, not just parathyroid adenoma, for which the surgical treatment would be en bloc resection of the parathyroid gland as well as the thyroid gland, adjoining fatty tissue, lymph nodes, and surrounding muscles in order to achieve the best chance for surgical cure. A tissue biopsy of the thyroid nodule was obtained. Rushed pathology of a fine needle aspiration of the 2.7 cm thyroid nodule was concerning for parathyroid carcinoma.

Calcitonin, cinacalcet, and zoledronic acid were added in succession, each with minimal improvement in hypercalcemia. For concurrent hyperthyroidism, she was also started on methimazole and propranolol in order to help prevent thyroid storm during possible surgical resection. Given incomplete administration of betamethasone one week before, the decision was made to administer a complete 2-dose course of betamethasone due to risk of premature delivery. Throughout the hospital course, continuous fetal monitoring showed reassuring fetal status. However, on the third day of medical management, the patient continued to have severe hypercalcemia refractory to medical management and fetal status began to deteriorate.

Failing medical management, and in the setting of worsening fetal status, the endocrine surgery team provided emergent surgical management. Unexpectedly, intraoperative frozen section of one of two pretracheal lymph nodes was consistent with metastatic papillary thyroid cancer. She underwent bilateral exploration of parathyroid glands, total thyroidectomy, right central neck dissection, and right cervical thymectomy. After completion of the procedure, intraoperative PTH level decreased to 53, consistent with complete removal of the parathyroid lesion. Final pathology showed a parathyroid adenoma with atypia as well as occult papillary thyroid carcinoma with metastasis to a single lymph node.

The patient tolerated the procedure well and was transported to an ICU bed for close monitoring. She was closely monitored and started on calcium carbonate, calcitriol, and levothyroxine. Her calcium normalized within 12 hours and her pancreatitis resolved. Fetal status was reassuring following the procedure.

On postoperative day 2, at 36 weeks and 2 days' gestation, the patient began to contract painfully. She declined trial of labor and underwent an uncomplicated repeat C-section. She delivered a female infant weighing 3400 g with APGARS 7 and 8. The infant's calcium levels were elevated immediately following delivery with an ionized calcium of 1.66, but 24 hours later she was hypocalcemic and required calcium supplementation. The infant's hypocalcemia improved and stabilized with improved feeding and she was discharged home at 5 weeks of age with vitamin D3 supplementation. The infant also received an ultrasound that was consistent with neonatal nephrocalcinosis likely secondary to longstanding intrauterine hypercalcemia that may take months or years to resolve. A follow-up renal ultrasound is planned at 6 months of age.

The patient, now status postsurgical removal of thyroid and parathyroid glands, was discharged home on lifelong calcitriol, calcium supplementation, and levothyroxine with close outpatient follow-up with endocrinology. She will also follow up with endocrinology for further monitoring and evaluation of her thyroid function and for discussion of radioiodine therapy versus surveillance for thyroid cancer surveillance.

## 3. Discussion

The differential diagnosis of hyperparathyroidism includes primary hyperparathyroidism, renal disease (secondary and tertiary hyperparathyroidism), and hypercalcemia of malignancy. Medications and other iatrogenic causes such as milk-alkali syndrome must also be considered. In this case, the patient was noted to be drinking several glasses of soy milk daily that may have been fortified with calcium. She also reported recent increasing use of calcium carbonate for epigastric pain which she attributed to GERD. However, significantly elevated PTH levels along with physical exam findings of palpable thyroid nodule pointed to the ultimate diagnosis.

Though primary hyperparathyroidism (PHPT) is relatively common in the general population (0.15%) and is three times more common in women, only 5–10% of these cases occur in women of childbearing age [7]. There are also racial differences in the incidence and prevalence of PHPT with rates among Hispanic women significantly lower than among black and white women (*p* < 0.0001). For women aged 20–29, the incidence of PHPT per 100,000 person-years is 12.1 for all women but is only 6.4 in Hispanic women. In the United States, the incidence is the highest among Asian women in this age group (25.6) and black women (23.7) and the lowest in white women (11.6) and other races (4.5) [8].

PHPT during pregnancy poses significant risks with maternal complications reported to be as high as 67–85%. PHPT leading to pancreatitis and hypercalcemic crisis can lead to significant maternal and fetal morbidity and mortality, with fetal and neonatal complication rates up to 45–80%, including spontaneous abortion (15%), intrauterine fetal demise (7%), neonatal hypocalcemia and tetany (22%), and neonatal death (11%) [2, 3, 9].

Early diagnosis can be difficult as many women are asymptomatic and many of the symptoms that do occur mimic those experienced in normal pregnancy including nausea, vomiting, polyuria, constipation, acid reflux, musculoskeletal pain, and fatigue. Maternal calcium absorption naturally increases during pregnancy; however, high calcium levels can be masked by a number of other physiologic adaptations during pregnancy, including hemodilution, gestational hypoalbuminemia, increased glomerular filtration rate, and transport across the placenta. For these reasons, it is important to check both serum calcium and ionized calcium levels [5, 9]. When serum calcium levels are high enough to cause hypercalcemic crisis (>15 mg/dL), the patients may have altered mental status, arrhythmias, electrolyte imbalances, renal impairment, and even coma and death [1, 3, 4, 7, 9]. The patient described in this case had corrected serum calcium levels as high as 16.9 mg/dL as well as electrolyte imbalances and EKG changes. However, the remainder of her symptoms was vague and nonspecific. Despite biochemical evidence of pancreatitis (amylase 334 U/L, lipase > 3000 U/L), she did not have the classic discomfort of pancreatitis.

The patient's history of pancreatitis in 2015 and nephrolithiasis in 2009 and evidence of both maternal and fetal nephrocalcinosis on imaging support longstanding hypercalcemia. This may explain why she did not present in florid hypercalcemic crisis despite her serum calcium levels and other electrolyte imbalances. Interestingly, her amniotic fluid index was within normal limits (15) on initial presentation but rapidly increased to an impressive 41.3 just one week later. Otherwise, unexplained fetal polyhydramnios has been associated with hypercalcemia, likely caused by fetal hypercalcemia and polyuria [2].

Neonatal nephrocalcinosis is a common finding among preterm babies and has been noted in 16–64% of low birth weight infants [10]. In these cases, nephrocalcinosis occurs as a result of an imbalance between stone-promoting and stone-inhibiting factors in premature kidneys. High calcium intake is a known risk factor for nephrocalcinosis in preterm neonates [11]. In this case, nephrocalcinosis was noted in utero as well as after delivery and was likely caused by maternal hypercalcemia. Neonatal nephrocalcinosis has been found in cases of maternal hypercalcemia resulting from hyperparathyroidism as well as other conditions such as vitamin-D-hydroxylase mutations. Clinical findings of hypercalcemia and nephrocalcinosis or a history of kidney stones should prompt further investigation of PTH and vitamin D levels [11].

In cases of nephrocalcinosis resulting from prematurity, spontaneous resolution has been shown in up to 85% of children in the first years of life. However, there is evidence of long-term effects on kidney function associated with neonatal nephrocalcinosis of prematurity and long-term follow-up of blood pressure and renal function is recommended [11]. In our patient, nephrocalcinosis secondary to prolonged exposure to maternal hypercalcemia is the most likely diagnosis and this may take months to years to resolve. Though the additional insult of prematurity may have added to this picture after delivery, without continued hypercalcemic exposure, chronic kidney disease is unlikely. Neonatal kidney function was good at the time of discharge and she will be followed closely as an outpatient.

The timing of surgical management of PHPT in pregnancy is somewhat controversial. The acute maternal presentation, fetal status, maternal medical and surgical history, and the patient's response to medical management should all be taken into careful consideration. Given the potential morbidity and poor fetal outcomes, initial reaction may be toward immediate delivery of the fetus. However, delivery can lead to sharp increase in maternal serum calcium levels as the calcium, which had previously been preferentially received by the fetus, now remains solely in maternal circulation. Additionally, the neonate may experience significant hypocalcemic crisis and tetany after delivery as its PTH levels are suppressed and previously maternally supplied calcium disappears [1, 3].

Despite this, in practice it may be difficult to postpone delivery confidently, particularly in the late preterm period. In less severe cases and in asymptomatic patients, medical management with the goal of normalization of serum calcium should precede delivery. There is growing agreement that severe hypercalcemia (persistent serum calcium > 11 mg/dL or worsening symptoms of hypercalcemia) should be treated surgically without an attempt at medical management. Surgery is considered the safest in the second trimester; however, there are now many reports of successful surgery in the third trimester without complications [12].

In this case, likely because of chronic and longstanding disease, both maternal and fetal status remained reassuring. Therefore, medical optimization of the patient's severe hypercalcemia was pursued in an attempt to prevent poor maternal and neonatal outcomes in the immediate postoperative period. Medical management was carefully guided by maternal status and continuous fetal monitoring. Neonatology, surgery, and endocrinology teams were consulted early and were involved in her ongoing care and management from the day of hospital admission. As the patient's hypercalcemia remained refractory to medical management and the fetal heart tracing became nonreassuring, the decision was made to proceed with surgical management of her hypercalcemia. The infant was delivered two days later in the setting of preterm labor. Ultimately, both mother and infant recovered well and were discharged in good condition.
